# CXXC5: A novel regulator and coordinator of TGF‐β, BMP and Wnt signaling

**DOI:** 10.1111/jcmm.14046

**Published:** 2018-11-27

**Authors:** Xiangyang Xiong, Shuo Tu, Jianbin Wang, Shiwen Luo, Xiaohua Yan

**Affiliations:** ^1^ Department of Biochemistry and Molecular Biology, School of Basic Medical Sciences Nanchang University Nanchang China; ^2^ School of Basic Medical Sciences Nanchang University Nanchang China; ^3^ Center for Experimental Medicine The First Affiliated Hospital of Nanchang University Nanchang China

**Keywords:** CXXC domain, CXXC5, signaling coordinator, transcription factor, tumour suppressor

## Abstract

CXXC5 is a member of the CXXC‐type zinc‐finger protein family. Proteins in this family play a pivotal role in epigenetic regulation by binding to unmethylated CpG islands in gene promoters through their characteristic CXXC domain. CXXC5 is a short protein (322 amino acids in length) that does not have any catalytic domain, but is able to bind to DNA and act as a transcription factor and epigenetic factor through protein‐protein interactions. Intriguingly, increasing evidence indicates that expression of the CXXC5 gene is controlled by multiple signaling pathways and a variety of transcription factors, positioning CXXC5 as an important signal integrator. In addition, CXXC5 is capable of regulating various signal transduction processes, including the TGF‐β, Wnt and ATM‐p53 pathways, thereby acting as a novel and crucial signaling coordinator. CXXC5 plays an important role in embryonic development and adult tissue homeostasis by regulating cell proliferation, differentiation and apoptosis. In keeping with these functions, aberrant expression or altered activity of CXXC5 has been shown to be involved in several human diseases including tumourigenesis. This review summarizes the current understanding of CXXC5 as a transcription factor and signaling regulator and coordinator.

## 1 INTRODUCTION

CXXC5, also known as retinoid‐inducible nuclear factor, is a member of the CXXC‐type zinc‐finger protein family. Members of this family are characterized by a specialized CXXC domain and include CXXC1/CFP1, CXXC2/KDM2B, TET1/3 and DNMT1, among others.^1^, ^2^, ^3^ The CXXC domain binds to unmethylated cytosine‐guanine (CpG) dinucleotides, especially those within short (500‐2000 bp) CpG islands and contiguous CpG‐rich DNA sequences in promoter regions. Intriguingly, these CpG islands are generally refractory to DNA methylation. Accordingly, many CXXC family members are involved in epigenetic regulation and therefore play pivotal roles in embryonic development, tissue homeostasis and pathological alterations.^1^, ^2^, ^4^

The CXXC5 protein, though much less understood, has been shown to act as a transcriptional regulator by binding directly to DNA.^5^, ^6^ Intriguingly, accumulating evidence indicates that CXXC5 gene expression is regulated by various secreted cytokines and intracellular transcription factors.^7^, ^8^, ^9^, ^10^ In addition, the CXXC5 protein is implicated in regulating and coordinating multiple signaling pathways including those initiated by transforming growth factor beta (TGF‐β), bone and morphogenetic proteins (BMPs), Wnt, ATM/p53 and others.^8^, ^10^, ^11^, ^12^ This review will first summarize the characteristics of the CXXC‐type zinc‐finger protein family and the role of CXXC5 as a transcriptional regulator. Next, we will give special attention to the mechanisms by which the CXXC5 protein regulates and coordinates different signaling pathways, thereby exerting multi‐faceted pathophysiological functions.

## 2 CXXC‐TYPE ZINC‐FINGER PROTEIN FAMILY

The combined pattern of DNA and histone modifications confers an epigenetic code that can profoundly affect both chromatin structure and gene expression.^13^ In vertebrates, DNA methylation mainly occurs on cytosine within the CpG dinucleotides, which are frequently found in mammalian gene promoters and are associated with a compacted chromatin structure and transcriptional repression.^14^ Previous studies have demonstrated that proteins harbouring methyl‐CpG‐binding domains (MBDs) play a significant role in this process.^14^, ^15^ However, as mentioned above, methylation‐resistant CpG islands are prevalent in many promoters, up to 60% of human gene promoters.^1^, ^2^, ^13^ It is reasonable to assume that these unmethylated CpG islands are also targeted by epigenetic factors and are associated with an open chromatin state and transcriptional activation. Indeed, the specialized CXXC‐type zinc‐finger domain has been shown to bind unmethylated CpG in vitro.^16^ Further, ChIP‐sequencing (ChIP‐Seq) analyses have demonstrated that some of the CXXC domain‐containing proteins are capable of binding to CpG at the genomic scale in various in vivo contexts.^1^, ^4^, ^17^

**Figure 1:**
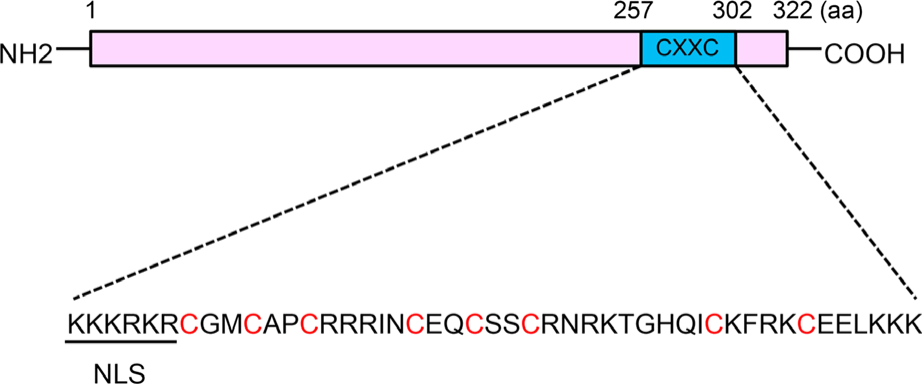
Diagrammatic presentation of the human CXXC5 protein. The sequence of the CXXC domain (amino acids 257‐302) is shown, and the nuclear localization sequence (NLS) (amino acids 257‐262) and the cysteine residues that are involved in the formation of zinc‐finger structures are underscored

Twelve CXXC zinc‐finger protein family members have been identified in mammalians (Table 1).^1^, ^2^, ^3^, ^18^ The CXXC‐type zinc‐finger domain is characterized by two conserved cysteine‐rich motifs (ie, CxxCxxCx_4‐5_CxxCxxC and CxxRxC, wherein x indicates residues other than cysteine), with three cysteines from the first motif and one from the latter together coordinating one Zn^2+^ ion, therefore forming two zinc‐finger structures.^2^, ^14^ However, the linker region between the two motifs is less conserved and classifies the family members into different subgroups. CFP1/CXXC1, KDM2A/CXXC8, KDM2B/CXXC2, FBXL19/CXXC11, DNMT1/CXXC9, MLL1/CXXC7, MLL2/CXXC10 and MBD1/CXXC3 share a conserved KFGG motif and fall into the same subgroup. Meanwhile, TET1/CXXC6, TET3, IDAX/CXXC4 and CXXC5 are shorter, lack the KFGG motif, and constitute a second subgroup. The majority of CXXC family members exhibit chromatin‐modifying activity due to the existence of functional domains. CXXC4 and CXXC5, though structurally similar, lack these types of domains.^11^, ^19^ The CXXC5 protein is 322 amino acids in length, has a molecular weight of 32.98 kDa, and contains a typical CXXC zinc‐finger domain (amino acids 257‐302) near the C‐terminus (Figure 1). A nuclear localization sequence (NLS) exists at the N‐terminus of the CXXC domain, between amino acids 257 and 262 (KKKRKR).^3^, ^11^, ^12^

**Table 1:** CXXC‐type zinc‐finger protein family in human

Member	Alias	Length (aa)	Catalytic activity
CXXC1	CGBP, CFP1	660	H3K4 methyltransferase
CXXC2	KDM2B, FBXL10	1336	H3K36 demethylase
CXXC3	MBD1	605	Interacts with and recruits the H3K9 methyltransferase SETDB1
CXXC4	IDAX	367	Interacts with and recruits TET2
CXXC5	RINF, WID	322	Interacts with and recruits TET2
CXXC6	TET1, LCX	2136	5‐methylcytosine dioxygenase
CXXC7	MLL1, KMT2A	3969	H3K4 methyltransferase
CXXC8	KDM2A, JHDM1a, FBXL11	1162	H3K36 demethylase
CXXC9	DNMT1	1616	DNA methyltransferase
CXXC10	MLL2, KMT2B	2715	H3K4 methyltransferase
CXXC11	FBXL19	694	A component of the Skp1‐Cullin‐F‐box family of E3 ubiquitin ligases
TET3		1660	5‐methylcytosine dioxygenase

## 3 CXXC5 ACTS AS A TRANSCRIPTION FACTOR AND/OR EPIGENETIC FACTOR

Although CXXC4 and CXXC5 do not have any catalytic domains, it is interesting that both have been found to interact with and recruit TET2, which functions in a similar way as TET1/3 to promote DNA demethylation but is unable to bind DNA directly due to the absence of the CXXC domain.^4^, ^5^ ChIP‐Seq analysis indicated that the CXXC domain of CXXC4 mainly interacts with unmethylated and CpG‐rich promoters throughout the genome in HEK293T cells.^4^ Intriguingly, CXXC4 interacts directly with the TET2 catalytic domain, leading to caspase‐mediated downregulation of TET2 protein levels in mouse embryonic stem cells (mESCs) and monocytic cells. Similarly, CXXC5 has recently been shown to recruit TET2. Genetic ablation of the CXXC5 gene resulted in a series of epigenetic alterations in the IRF7 gene region, including decreased levels of promoter CpG methylation as well as H3K4m3, H3K36m3, H3K27ac and H4ac histone modifications in plasmacytoid dendritic cells, thus contributing to TLR7/9‐ and virus‐induced IFN responses.^5^

Several other reports have provided additional evidence that CXXC5 could act as a transcription factor and/or epigenetic factor by binding to DNA via its CXXC domain (Table 2). In neural stem cells, Wnt3a‐stimulated CXXC5 directly interacts with the promoter region (−720 to −511 bp) and promotes the expression of the myelin basic protein gene, which is a molecular marker for oligodendrocyte differentiation.^20^ In endothelial cells, the CXXC5 protein interacts with the Flk‐1 promoter through its CXXC domain, activating Flk‐1 expression and promoting endothelial cell differentiation, migration and vessel formation.^7^ In C2C12 myoblasts, CXXC5 is able to increase the activities of gene promoters that drive the expression of muscle creatine kinase, minimal thymidine kinase and myosin heavy chain, thus promoting skeletal muscle differentiation.^21^

**Table 2:** Genes directly regulated by CXXC5

Gene	Regulation by CXXC5	Function	References
IRF7	Up	Contributes to TLR7/9‐ and virus‐induced IFN responses	5
MBP	Up	Promotes differentiation of neural stem cells into oligodendrocytes	20
Flk‐1	Up	Promotes endothelial cell differentiation, migration and vessel formation	7
MCK, MHC	Up	Promotes skeletal muscle differentiation	21
Cd40L	Down	Inhibits differentiation into helper‐lineage T cells	22
COX4I2	Down	Inhibits energy production in hypoxic conditions	6

MBP, myelin basic protein; MCK, muscle creatine kinase; MHC, myosin heavy chain.

In other contexts, CXXC5 may repress gene expression. For instance, in CD8+ Th cells, CXXC5 could associate with the histone‐lysine N‐methyltransferase SUV39H1, inducing histone H3K9 methylation in the Cd40L gene promoter region and inhibiting CD40 ligand expression.^22^ COX4 is one subunit of the Cytochrome c oxidase (COX) complex. An oxygen responsive element (ORE) has been identified in the promoter region of the COX4I2 gene isoform. In mammalian cell lines, CXXC5 interacts with the ORE to inhibit COX4I2 expression under hypoxic conditions.^6^ Together, these observations indicate that CXXC5 acts as a transcription factor and/or epigenetic factor to promote or repress gene expression, depending on cell types and contexts (Table 2).

## 4 CXXC5 FUNCTIONS AS A SIGNALING REGULATOR AND MEDIATOR

### 4.1 CXXC5 in TGF‐β/BMP signaling

Transforming growth factor‐β is a multi‐functional peptide that plays a critical role in regulating various cellular functions and exerts a diverse array of physiological functions. Deregulation of TGF‐β signaling has been connected with several major human diseases, including developmental defects, tumourigenesis, cardiovascular disorders and metabolic syndromes.^23^, ^24^, ^25^, ^26^, ^27^, ^28^, ^29^ TGF‐β is the founding member of the TGF‐β cytokine family, which includes TGF‐β itself, Activin, Nodal, Lefty, BMPs, GDFs (growth and differentiation factors), AMH (anti‐Müllerian hormone) and others. These peptides are roughly grouped into two subclasses based on structural similarities and signaling properties. TGF‐β, Activin, Nodal and Lefty fall into the TGF‐β subclass, while the majority of BMPs, GDFs and AMH constitute the BMP subclass. All of these cytokines transduce signals by binding to serine/threonine kinase receptors on the cell membrane, leading to the phosphorylation and activation of Smad proteins.^30^, ^31^, ^32^, ^33^, ^34^, ^35^ In total, there are eight Smad proteins in mammalians, which are functionally classified into three subgroups: R‐Smads (receptor‐regulated Smads), Co‐Smad (common Smad, Smad4) and inhibitory Smads (I‐Smads, Smad6 and Smad7).^33^, ^36^ TGF‐β subfamily cytokines utilize Smad2 and Smad3 as R‐Smads, while BMPs activate Smad1/5/8. Activated R‐Smads form a complex with Smad4, and together they translocate into the nucleus to regulate target gene transcription in collaboration with other transcription factors or co‐factors.^37^, ^38^ In addition, TGF‐β is capable of activating other signaling pathways mediated by PI3K/Akt, MAPKs (EKR1/2, JNKs and p38), PAK2 and Cdc42/RhoA GTPases, among others.^39^, ^40^, ^41^

One major cellular response to cytokine stimulation is transcriptional alteration. Target gene identification and their functional analyses are crucial for understanding the context‐dependent biological roles of specific cytokines, including TGF‐β.^38^, ^42^ With this regard, transcriptional expression of the CXXC5 gene is induced by BMP4 in embryonic telencephalic neural stem cells, mESC‐derived endothelial cells and human umbilical vein endothelial cells.^7^, ^11^ Functionally, CXXC5 mediates BMP4‐induced repression of Wnt signaling in neural stem cells, as well as endothelial cell migration and vessel formation. In addition, we have previously reported that CXXC5 is a target gene and functional mediator of TGF‐β in hepatocellular carcinoma (HCC) cells, contributing to the tumour‐suppressive functions of TGF‐β by promoting cell cycle arrest and apoptosis.^10^

Another important point to understand the pleiotropic nature of TGF‐β is the spatiotemporal regulatory mechanisms of the signal transduction process.^31^, ^34^, ^43^ Feedback regulation plays a pivotal role, fine‐tuning signaling robustness and duration.^32^, ^44^ With respect to it, the two inhibitory Smads have been shown critical in controlling TGF‐β/BMP signaling through negative feedback mechanisms.^36^, ^45^, ^46^, ^47^, ^48^ We found that overexpression of the CXXC5 protein in both HCC cells and normal hepatocyte cell lines promotes TGF‐β‐induced luciferase reporter expression, while knockdown of CXXC5 gene ameliorates it. Moreover, transcriptional profiling indicates that depletion of CXXC5 in Hep3B HCC cells is able to attenuate the expression of a significant portion of TGF‐β target genes, suggesting that CXXC5 is a novel positive feedback regulator of TGF‐β signaling.^10^ Moreover, the CXXC5 protein associates with the histone deacetylase HDAC1 in HCC cells and competes for Smad2/3 binding, therefore alleviating HDAC1‐mediated signaling inhibition. Intriguingly, CXXC5 was also suggested to interact with Smad2/3 and Smad4 to promote TNF‐α‐induced apoptosis of primary cortical neurons and normal heart development and function in zebrafish.^49^, ^50^

### 4.2 CXXC5 in Wnt/β‐catenin signaling

The Wnt family of cytokines plays a critical role in embryonic development and adult tissue homeostasis in multicellular animals.^51^, ^52^, ^53^ In the Wnt/β‐catenin pathway, the β‐catenin protein is sequestered in a multi‐protein complex in the absence of ligand stimulation. This complex is comprised of the scaffold proteins Axin and APC as well as two constitutively active serine‐threonine protein kinases, GSK3β and CK1. Sequestration of β‐catenin leads to its phosphorylation and subsequent poly‐ubiquitination and degradation by the F‐box/WD repeat protein β‐TrCP, part of an E3 ubiquitin ligase complex.^51^, ^52^, ^54^, ^55^ Upon ligand binding, the seven‐pass transmembrane receptor Frizzled (Fzd) and the single‐pass receptor LRP5/6 undergo conformational changes and phosphorylation. This is followed by recruitment of the Axin and Dvl proteins and breakdown of the destruction complex, leading to release and stabilization of β‐catenin, which then enters the nucleus and acts as a transcriptional activator for TCF/LEF transcription factors, thus controlling the expression of a diverse assay of target genes. In addition to this canonical signaling, some Wnt ligands are capable of initiating signaling pathways independent of β‐catenin, including the PCP pathway and those involving release of calcium ions or activation of JNK, Src or other molecules.^56^, ^57^

Not surprisingly, the Wnt/β‐catenin pathway is also subject to intense regulation.^54^, ^55^, ^58^ Within the CXXC‐type zinc‐finger protein family, CXXC4 has been shown to interact with Dvl, resulting in inhibition of β‐catenin signaling.^19^, ^59^, ^60^ Similarly, CXXC5 is also able to inhibit β‐catenin signaling by interacting with the PDZ domain of the Dvl protein via its C‐terminal CXXC domain, thereby inhibiting Wnt3a‐induced osteoblast differentiation, bone formation, cutaneous wound healing, in addition to disturbing embryonic kidney development.^8^, ^9^, ^61^ These results suggest that CXXC4 and CXXC5 not only share structural similarities but are also functionally conserved in regulating Wnt signaling. Intriguingly, Wnt3a is able to induce CXXC5 expression in pre‐osteoblast MC3T3E1 cells, human dermal fibroblasts and neural stem cells, making CXXC5 a negative feedback regulator.^8^, ^9^, ^20^

### 4.3 CXXC5 in other signaling pathways

The p53 tumour suppressor acts as a major defence against cancer by inducing DNA repair, cell cycle arrest, senescence or apoptosis in response to diverse cellular stresses.^62^ The ataxia telangiectasia mutated (ATM) protein kinase, a member of the PI3/PI4‐kinase family, phosphorylates p53 upon DNA damage, leading to stabilization and activation of the p53 protein, predisposing it for DNA damage repair.^63^, ^64^ Interestingly, the CXXC5 protein has been found to interact with ATM and is required for DNA damage‐induced ATM phosphorylation, p53 stabilization and activation, and subsequent DNA damage response.^12^

1α,25‐dihydroxyvitamin D3 (1,25D) is the active hormonal metabolite of vitamin D. It incites a variety of biological functions by binding the vitamin D receptor (VDR), thus stimulating its transcriptional activity.^65^ In a yeast two‐hybrid screening, CXXC5 was identified as a novel VDR‐interacting protein, promoting VDR‐mediated transcription from selected Vitamin D‐responsive DNA elements (VDREs).^66^ Given that VDR has been shown to inhibit Wnt signaling by associating with β‐catenin and restricting its nuclear localization,^67^, ^68^ an interesting question to consider is whether VDR and CXXC5 might cooperate with one another to restrain Wnt signaling. FoxL2 is a transcription factor belonging to the large family of winged‐helix forkhead transcription factors. This protein has been implicated in numerous cellular processes including apoptosis, cell cycle control, steroidogenesis and reactive oxygen species (ROS) detoxification.^69^ Both CXXC4 and CXXC5 can interact with FoxL2, attenuating its transcriptional activity in luciferase reporter assays while facilitating its pro‐apoptosis activity via unknown mechanisms.^70^ The known interacting partners of CXXC5 are summarized in Table 3.

**Table 3:** Interacting partners of CXXC5 protein

Interacting partner	Interaction site	Functional consequence of the interaction	References
Dishevelled	Cytoplasm	Inhibits Wnt/β‐catenin signaling	8, 9, 11, 61
HDAC1	Nd.	Removes HDAC1 from Smad2/3 and promotes TGF‐β signaling	10
Smad2/3/4	Predominantly nucleus	Mediates TNF‐α‐induced apoptosis and regulates zebrafish heart development	49, 50
ATM	Mainly nucleus	Contributes to p53 activation and DNA damage response	12
VDR	Nd.	Stimulates VDR transcriptional activity	66
SUV39H1	Nd.	Represses CD40 ligand expression in CD8+ cytotoxic T cells	22
RBPJ	Nd.	Inhibits COX4I2 gene transcription	6
Tet2	Nucleus	Contributes to IFN response by activating IRF7 expression	5

Nd., not determined; ATM, ataxia telangiectasia mutated; VDR, vitamin D receptor; TGF‐β, transforming growth factor beta.

## 5 CXXC5 FUNCTIONS AT THE CROSSROADS OF MULTIPLE SIGNALING PATHWAYS

The CXXC5 gene is evolutionarily conserved from Xenopus and zebrafish to mammals. It is located on chromosome 5q31.3 in human, spans 35.5 kb and contains 4 exons, with an mRNA product of approximately 1.4 kb.^71^, ^72^ CXXC5 is ubiquitously expressed across human development and adult tissues, though its expression level varies.^49^ Another study examined CXXC5 expression in human tissues and found that it is highly expressed in placenta, heart and kidney, and moderately expressed in liver, lung and testis.^3^

As mentioned above, the expression of the CXXC5 gene is transcriptionally induced by TGF‐β, BMP4 and Wnt3a, which rely on Smad proteins and β‐catenin respectively.^7^, ^9^, ^10^ In turn, the CXXC5 protein is able to inhibit Wnt signaling or facilitate TGF‐β signaling, forming distinct feedback regulatory loops. Considering that the three pathways often crosstalk with one another in different contexts including embryonic development, homeostatic adult tissues and pathological conditions,^73^, ^74^ it is conceivable that CXXC5 could be a novel crosstalk mediator of these pathways. Indeed, BMP4 induces CXXC5 gene expression in neural stem cells, to antagonize Wnt/β‐catenin signaling and its target gene expression.^11^ In addition, CXXC5 and BMP4 expression are closely correlated in the dorsal telencephalon of mouse forebrain, just adjacent to Wnt3a expression, strongly suggesting an in vivo functional link.^11^

Several transcription factors have been shown to bind to the CXXC5 promoter or enhancer, thereby regulating its transcription (Table 4). The zinc‐finger transcription factor Wilms tumour 1 has been shown to activate CXXC5 gene transcription in mammalian cell lines by directly interacting with DNA sequences in the upstream enhancer region.^61^ GATA2 is a DNA‐binding transcription factor that is important for hematopoietic stem cell proliferation. Mutations in the GATA2 gene have been associated with familial myelodysplastic syndrome and acute myeloid leukaemia (AML).^75^, ^76^ Interestingly, GATA2 can bind to the promoter of the CXXC5 gene, increasing its expression. Accordingly, CXXC5 mRNA levels have been found significantly downregulated in cells from AML patients with GATA2 mutations.^71^ In addition, CXXC5 can be silenced by promoter hypermethylation in prostate cancer cells.^77^ Therefore, epigenetic alterations or transcription factor mutations could contribute to aberrant CXXC5 expression in cancer. KANK1 is a potential tumour suppressor gene and is downregulated in more than half of human malignant peripheral nerve sheath tumours (MPNSTs).^78^ Overexpression of KANK1 could induce CXXC5 expression to promote cell apoptosis in MPNSTs.^79^ The retinoic acid receptor (RARα) acts as a transcription factor when bound to all‐trans retinoic acid, facilitating terminal maturation of leukaemic blasts.^80^ The CXXC5 promoter contains a retinoid‐responsive element at −3116 bp. Ligand‐bound RARα directly binds to this regulatory element in NB4 acute promyelocytic leukaemia cells, promoting CXXC5 gene expression and leukaemia cell differentiation.^3^ 17β‐estradiol (E2)‐ERα signaling plays a critical role in both physiological functions and malignant transformation of breast tissue.^81^ E2‐ERα is able to interact with an oestrogen‐responsive element (ERE) (GGTCAnnnTGACC) in the CXXC5 gene promoter, inducing its gene expression in breast cancer cells.^82^ Conversely, ThPOK, a zinc‐finger family transcription factor encoded by the Zbtb7b gene, represses CXXC5 gene expression in CD8+ cytotoxic T cells.^22^ Interestingly, NUDT21 (Nudix Hydrolase 21), one subunit of a cleavage factor required for 3’ RNA cleavage and polyadenylation, was found to repress CXXC5 expression by inducing alternative polyadenylation in liver cancer cells.^83^

**Table 4:** Transcriptional regulation of the CXXC5 gene

Cytokine or transcription factor	CXXC5 expression	Cell type and context	References
TGF‐β	Up	Hepatocellular carcinoma cells and normal liver cells	10
BMP4	Up	Embryonic neural stem cells, endothelial cells and HUVECs	7, 11
Wnt3a/β‐catenin	Up	Pre‐osteoblast MC3T3E1 cells, human dermal fibroblasts and neural stem cells	8, 9, 20
E2/ERα	Up	Breast cancer cells	82
Retinoid/RARα	Up	NB4 myeloid cells	3
WT1	Up	Zebrafish embryonic kidney development	61
KANK1	Up	Embryonic kidney	79
GATA2	Up	Acute myeloid leukemia	71
ThPOK	Down	CD8+ cytotoxic T cells	22
NUDT21	Down	Liver cancer cells	83

TGF‐β, transforming growth factor beta; BMPs, bone and morphogenetic proteins.

**Figure 2:**
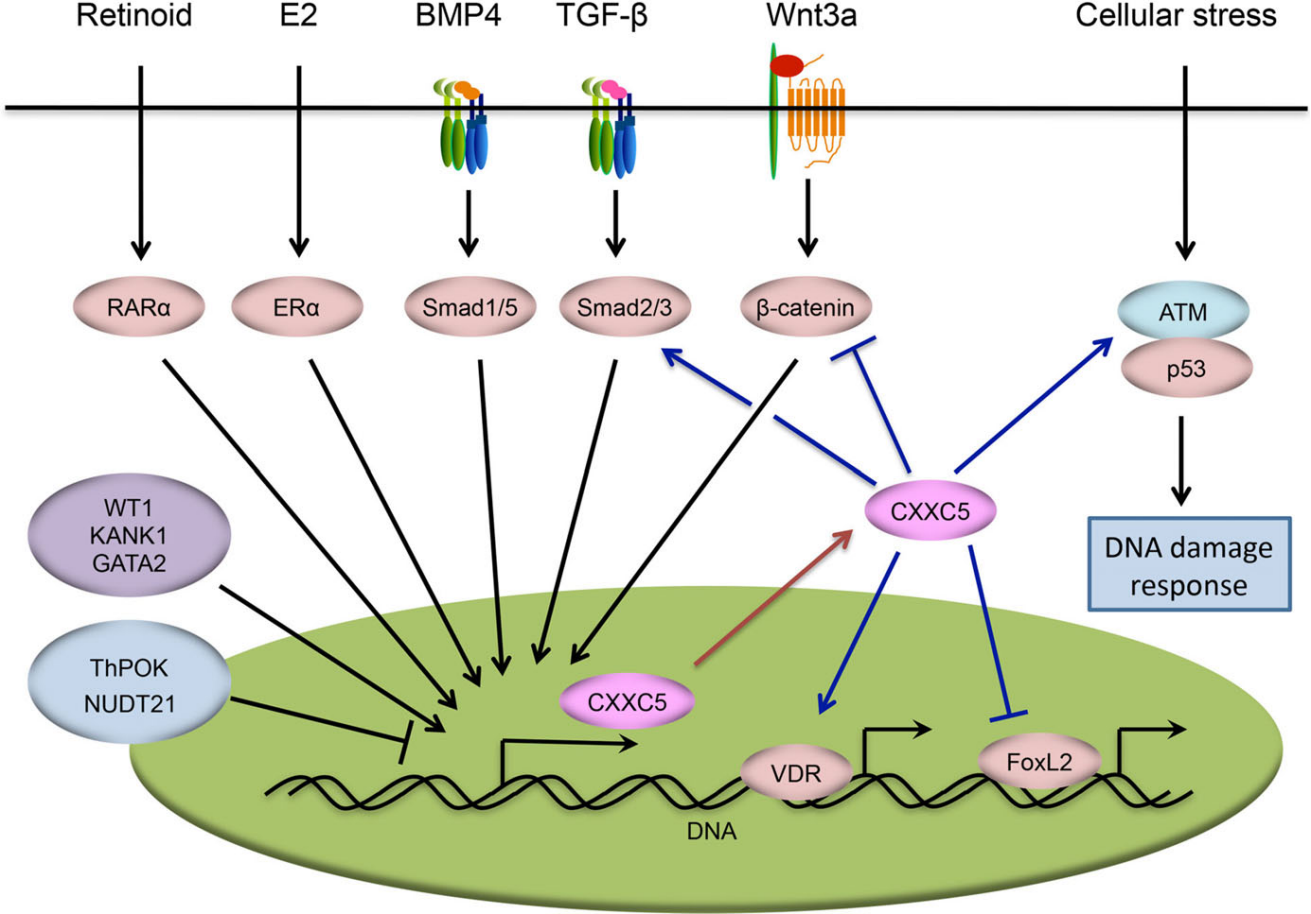
CXXC5 is an integrator and coordinator of cellular signaling networks. TGF‐β, BMP4 and Wnt3a induce CXXC5 gene expression in different contexts. The protein product of this gene can in turn inhibit Wnt signaling or facilitate TGF‐β signaling, forming distinct regulatory feedback loops. The expression of CXXC5 is also transcriptionally induced by Retinoid/RARα and E2/ERα signaling as well as by some transcription factors, including WT1, KANK1 and GATA2. Conversely, the transcription factor ThPOK and the RNA cleavage factor NUDT21 are capable of repressing CXXC5 gene expression. In addition to TGF‐β and Wnt signaling, CXXC5 promotes ATM‐mediated activation of the p53 protein and DNA damage response. CXXC5 also enhances the transcriptional activity of VDR and ameliorates that of FoxL2. TGF‐β, transforming growth factor beta; BMPs, bone and morphogenetic proteins; ATM, ataxia telangiectasia mutated; VDR, vitamin D receptor

Together, different cytokines and transcription factors converge on the CXXC5 gene to regulate its expression, suggesting that CXXC5 may act as an important downstream integrator and mediator of various signal inputs. Taking into account its ability to fine‐tune various signal transduction processes, CXXC5 is likely to function as a crucial coordinator of multiple cellular signaling pathways (Figure 2).

## 6 PATHOPHYSIOLOGICAL FUNCTIONS OF CXXC5

Considering its roles in regulating gene expression and coordinating cellular signaling, it is not surprising that CXXC5 regulates a diverse array of cellular events including cell proliferation, differentiation and apoptosis in developmental processes, adult tissue maintenance and pathological circumstances.

With respect to cell differentiation, retinoid‐induced CXXC5 has been shown to play a role in normal primary leukaemia cell differentiation and tumoral myelopoiesis.^3^ BMP4‐activated CXXC5 promotes the differentiation of mESCs into endothelial cells.^7^ In mice, both BMP4‐ and Wnt‐induced CXXC5 can promote the differentiation of neural stem cells into oligodendrocytes, contributing to brain development and maturation.^11^, ^20^ In addition, CXXC5 facilitates skeletal myogenic differentiation in vitro by promoting the expression of related genes.^21^ Conversely, CXXC5 inhibits Wnt‐induced myofibroblast differentiation of dermal fibroblasts in cutaneous wound healing, and osteoblast differentiation and bone formation in mice.^8^, ^9^ Interestingly, a peptide that competitively inhibits the CXXC5‐Dvl interaction is capable of alleviating CXXC5‐mediated inhibition of Wnt signaling and accelerating both osteoblast differentiation and cutaneous wound healing.^8^, ^9^ In CD8+ cytotoxic T cells, CXXC5 represses CD40L expression, thus preventing the differentiation of helper‐lineage T cells.^22^ Importantly, gene‐targeting studies in mice have verified the in vivo functions of CXXC5 in regulating cell differentiation.^7^, ^8^, ^9^, ^20^

CXXC5 is involved in regulating cell proliferation and death in various cell types. In primary leukaemia cells, CXXC5 not only promotes differentiation, as mentioned above, but it also contributes to retinoid‐induced cell cycle arrest and cell death.^3^ CXXC5 is capable of promoting cell cycle arrest, DNA repair or apoptosis by activating the ATM‐p53 signaling axis.^12^ In addition, CXXC5 associates with FoxL2 to enhance its pro‐apoptotic activity in mammalian cells,^70^ and may mediate TNF‐α‐induced apoptosis of primary cortical neurons by associating with Smad proteins.^49^

**Figure 3:**
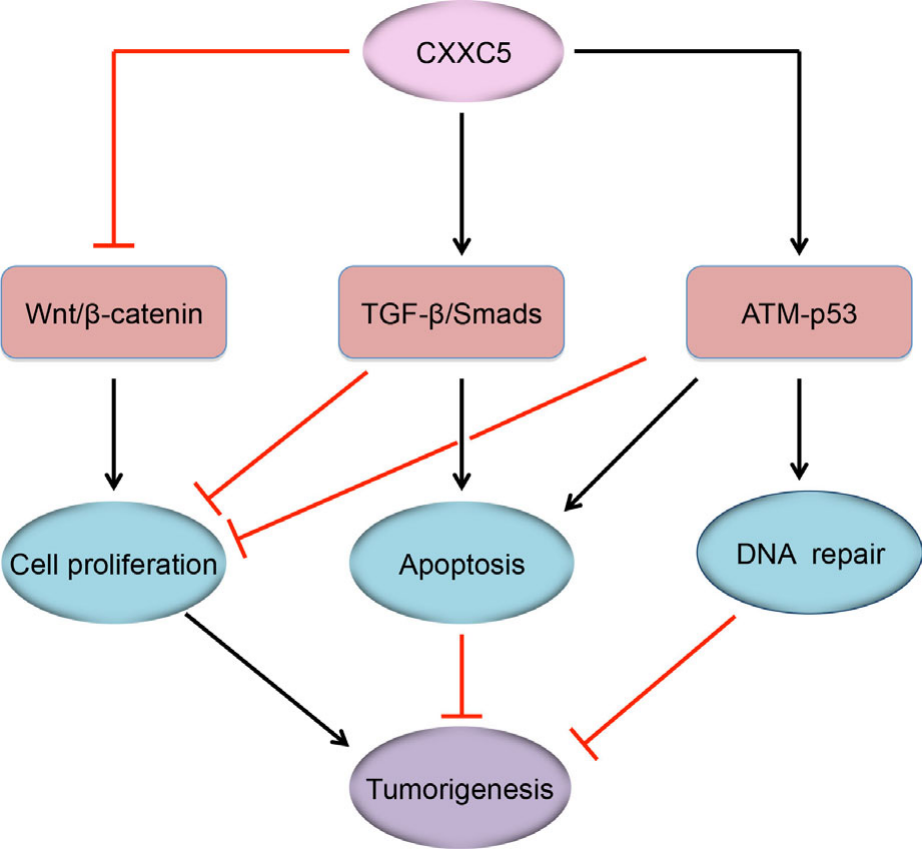
The tumour‐suppressive roles of CXXC5. CXXC5 is able to inhibit leukemia cell proliferation and tumourigenesis by inhibiting Wnt/β‐catenin signaling or facilitating ATM‐p53‐induced DNA damage response. In hepatocellular carcinoma cells, CXXC5 may act as a tumor suppressor by enhancing TGF‐β‐induced cell cycle arrest and apoptosis. TGF‐β, transforming growth factor beta; ATM, ataxia telangiectasia mutated

Aberrant expression or altered activity of CXXC5 is closely linked to multiple human diseases, especially tumourigenesis (Figure 3). The CXXC5 gene locates in a chromosomal DNA region that is frequently deleted in myeloid leukaemia (AML and myelodysplasia).^3^ Indeed, CXXC5 is often downregulated in clinical AML samples and may serve as an independent prognostic factor in patients.^71^, ^72^ CXXC5 downregulation leads to augmentation of Wnt signaling and impairment of DNA damage‐induced and p53‐dependent cell cycle arrest.^71^ We found that CXXC5 is also downregulated in the majority of HCC tissue samples, leading to breakdown of CXXC5‐mediated positive feedback regulation of TGF‐β signaling and attenuation of its anti‐proliferative effects.^10^ In addition, CXXC5 is involved in KANK1‐induced cell growth inhibition and apoptosis in MPNSTs.^79^ However, it has also been shown that CXXC5 is overexpressed in ER+ breast cancer and associated with poor prognosis in patients.^84^ This is in agreement with the observation that CXXC5 is upregulated by E2‐ERα.^82^ Together, these findings suggest that the role of CXXC5 in cancer initiation and progression could rely on cancer types.

CXXC5 has also been observed to play a role in some other pathological processes. Aberrant Wnt signaling has been found to be associated with hair loss (alopecia) and anabolic osteoporosis. A previous study demonstrated that expression of CXXC5 is upregulated and is inversely correlated with that of β‐catenin in both haired and bald individuals.^85^ In mice, CXXC5 knockout or competitive peptide‐mediated disruption of the CXXC5‐Dvl interaction restores Wnt signaling and accelerates hair regrowth and wound‐induced hair follicle neogenesis.^85^ Similarly, targeting the CXXC5‐Dvl interaction with small molecules has been suggested as a new therapeutic approach for the treatment of anabolic osteoporosis.^86^, ^87^

## 7 PERSPECTIVES

CXXC5 shares a conserved CXXC domain with other CXXC‐type zinc‐finger protein family members, which utilize this domain to recognize unmethylated CpG dinucleotides, especially those within CpG islands in gene promoters. Therefore, the majority of the proteins in this family, including CXXC5, act as transcription factors and epigenetic regulators.^1^, ^2^ Intriguingly, CXXC5 is emerging as a pivotal regulator of cellular signaling networks, as it not only receives various signal inputs and changes its expression accordingly, but also regulates and coordinates different signaling pathways. Although much progress has been made in understanding the molecular biology and pathophysiology of CXXC5, several interesting and important questions remain unanswered.

First, although CXXC5 is capable of associating with unmethylated CpG islands via its CXXC domain and regulating the expression of certain genes, the specific genomic sites at which CXXC5 binds to in a given cell type or context remain to be determined.

Second, CXXC5 is regulated by various cytokines and transcription factors and in turn regulates the intensity and duration of several cellular pathways. However, it is unknown whether and how the different signals converge on CXXC5 expression and how CXXC5 coordinates various signaling pathways in a certain circumstance.

Third, the CXXC domain plays a crucial role in CXXC5‐mediated regulation of gene expression or signal transduction. However, as this domain is conserved in other CXXC‐type zinc‐finger proteins, it remains unclear how the functional specificity of CXXC5 is achieved and what functions the amino acids out of the CXXC domain may exert.

Fourth, targeting of CXXC5, for example through inhibition of its interaction with the Dvl protein using competitive peptides or small molecules, is emerging as a promising means of therapeutic intervention in animal models. Given that aberrant CXXC5 expression is involved in various human diseases, especially cancer, it is reasonable to believe that targeting CXXC5 could be an effective clinical approach to treat these diseases.

## 8 COMPLIANCE WITH ETHICS GUIDELINES

The authors declare that they have no conflicts of interest. This is a review of previous studies and it does not contain any original human or animal studies performed by any of the authors.
